# Operational considerations for the management of non-communicable diseases in humanitarian emergencies

**DOI:** 10.1186/s13031-021-00345-w

**Published:** 2021-02-25

**Authors:** F. Jacquerioz Bausch, D. Beran, H. Hering, P. Boulle, F. Chappuis, C. Dromer, P. Saaristo, S. Aebischer Perone

**Affiliations:** 1 Division of Tropical and Humanitarian Medicine, Geneva University Hospitals, Rue Michel-Servet 1, 1206 Geneva, Switzerland; 2 Faculty of Medicine, University of Geneva, Rue Gabrielle-Perret-Gentil 6, 1205 Geneva, Switzerland; 3 United Nations High Commissioner for Refugees, Rue de Montbrillant 94, 1202 Geneva, Switzerland; 4 Médecins Sans Frontières, Rue de Lausanne 78, 1202 Geneva, Switzerland; 5 Health Unit, International Committee of the Red Cross (ICRC), 19, avenue de la Paix, 1202 Geneva, Switzerland; 6 International Federation of the Red Cross, Chemin des Crêts 17, 1209 Geneva, Switzerland

**Keywords:** Non-communicable diseases, Guidelines, Health services, Conflicts, Crises, Humanitarian emergencies, Continuum of care, Ethics, Humanitarian agencies

## Abstract

Non-communicable diseases (NCD) represent an increasing global challenge with the majority of mortality occurring in low- and middle-income countries (LMICs). Concurrently, many humanitarian crises occur in these countries and the number of displaced persons, either refugees or internally displaced, has reached the highest level in history. Until recently NCDs in humanitarian contexts were a neglected issue, but this is changing. Humanitarian actors are now increasingly integrating NCD care in their activities and recognizing the need to harmonize and enhance NCD management in humanitarian crises. However, there is a lack of a standardized response during operations as well as a lack of evidence-based NCD management guidelines in humanitarian settings. An informal working group on NCDs in humanitarian settings, formed by members of the World Health Organization, Médecins Sans Frontières, the International Committee of the Red Cross, the International Federation of the Red Cross and others, and led by the United Nations High Commissioner for Refugees, teamed up with the University of Geneva and Geneva University Hospitals to develop operational considerations for NCDs in humanitarian settings. This paper presents these considerations, aiming at ensuring appropriate planning, management and care for NCD-affected persons during the different stages of humanitarian emergencies. Key components include access to treatment, continuity of care including referral pathways, therapeutic patient education/patient self-management, community engagement and health promotion. In order to implement these components, a standardized approach will support a consistent response, and should be based on an ethical foundation to ensure that the “do no harm” principle is upheld. Advocacy supported by evidence is important to generate visibility and resource allocation for NCDs. Only a collaborative approach of all actors involved in NCD management will allow the spectrum of needs and continuum of care for persons affected by NCDs to be properly addressed in humanitarian programmes.

## Background

The increasing burden of non-communicable diseases (NCD) is a global challenge causing, according to the World Health Organization (WHO), 71% of global deaths (41 million) in 2018, with 85% of the deaths in people between the ages of 30 and 69 years occurring in low- and middle-income countries (LMICs) [1]. NCDs and humanitarian crises often co-occur in LMICs, providing additional challenges for the management of NCDs in these settings where weak health systems collide with the challenges of a humanitarian response. The number of displaced persons (either refugees or internally displaced) has reached the highest level in history, estimated at more than 70 million people [2]. Until recently NCDs in humanitarian contexts were a neglected issue [3, 4]. Humanitarian contexts are changing. Protracted crises are now also impacting higher income regions, such as the Middle East, and more displaced persons are settling in urban areas rather than traditional camp settings [5, 6]. This results in impacted populations being more likely to have pre-existing NCDs. All these factors influence the burden of NCDs seen in humanitarian crises as well as the approaches needed to address NCD-related health needs.

The WHO’s Global Action Plan (GAP) for the Prevention and Control of NCDs for 2013–2020 includes recommendations regarding NCDs in the humanitarian response [7]. This document states “it must be ensured that the use of the services does not expose the users to financial hardship, including in cases of ensuring the continuity of care in the aftermath of emergencies and disasters.” The recommendations also highlight the need to “improve the availability of life-saving technologies and essential medicines for managing NCDs in the initial phase of an emergency response.” Continuity of care in this context refers to: “access to comprehensive services and interventions that address the health needs and the well-being of a person, from the identification of a health condition until the recovery of a functional state consistent with the context” [8].

Given the relatively recent focus on NCDs during emergencies, humanitarian actors recognized the need to harmonize and enhance NCD management in humanitarian crises. Indeed, the humanitarian community has increasingly addressed the needs for NCD care in their activities [9] and developed organization-specific programmatic and clinical guidance. However, there is a lack of a standardized response during operations [3, 10, 11], which is essential to guarantee continuity of services to people in crisis settings, as well as a lack of evidence-based NCD management guidelines in humanitarian settings [12, 13]. WHO’s Package of Essential Noncommunicable Disease Interventions (PEN), provides a basis for NCD care in LMICs, but needs to be adapted to humanitarian settings to address the additional challenges of NCD care during crisis, which include disruption of health care services due to damaged and destroyed health facilities, lack of health care workers, difficulties in access to health facilities due to security constraints or damaged infrastructure. Therefore, a common approach of all actors in the field is needed to answer to the needs of patients with NCDs in crisis settings. The aim of this paper is to present operational considerations, aiming at ensuring appropriate planning, management and care for NCD-affected persons during humanitarian emergencies.

## Approach adopted

The informal working group on NCDs in humanitarian settings, formed by members of the WHO, Médecins Sans Frontières (MSF), the International Committee of the Red Cross (ICRC), the International Federation of the Red Cross (IFRC), the International Rescue Committee (IRC) and others, and led by the United Nations High Commissioner for Refugees (UNHCR), teamed up with the University of Geneva and Geneva University Hospitals in order to develop the operational considerations needed to address NCDs in humanitarian settings. This was done by applying a modified nominal group approach through a series of face to face meetings and email exchanges, informed by expert knowledge and relevant literature [14].

## Operational considerations

Humanitarian response interventions are usually prioritized according to needs and resources. NCDs encompass a spectrum of diseases and care requirements, and a prioritization of NCD services/interventions are required based on the available resources (human, financial, etc.) and on the context [15]. The list of conditions considered as a priority is based on feasibility during an acute humanitarian crisis, burden of disease and demand, avoidable premature deaths as defined in the priority NCDs of the WHO [7], those which have severe consequences if left untreated [16], and pre-crisis availability and capacity of the health system [17]*.*

### Priority NCDs and prioritization of care delivery in humanitarian settings

The priority NCDs to be included in humanitarian responses, as defined by the informal working group, are cardiovascular diseases (including heart failure of any etiology and coronary heart disease), high blood pressure, asthma, chronic obstructive pulmonary diseases (COPD), epilepsy and diabetes. In addition, long-term complications of NCDs, such as disability, stroke or amputation from diabetes should be considered (See Table 1). In accordance with the public health approach in humanitarian crises, some services/interventions need to be temporarily deprioritized in the initial phase of the response and integrated later on. Other interventions might not be relevant or feasible at the health facility where care is provided. For example, provision of cancer chemotherapy or dialysis for end-stage renal disease is rarely possible [19] and cannot be sustained by agencies with short-term mandates. These patients should be referred to second and third level hospitals wherever possible, as they require resource-intensive diagnostic and treatment means. Moreover, by enrolling patients into long-term programs and life-long therapies such as cancer care and renal dialysis comes financial and ethical responsibility [17]. However, supportive/palliative components of care should be provided at every stage of the response [20].

**Table 1:** **Pakistan, Muzzafarabad **: to illustrate choice of priority condition, integrated approach, health promotion and community engagement

The prevalence of diabetes in Pakistan is estimated by the WHO to be 9.8% [18]. In 2016 more than 15% of lower limb amputations followed-up at the physical rehabilitation center of Muzzafarabad, Pakistan were related to diabetes complications.	
Recognising the burden of diabetic foot problems which could be prevented, the health directorate of Muzzafarabad asked the ICRC in 2017 to support them in decentralisation of diabetes care from tertiary to the PHC level, with links to the community and referral to secondary and tertiary care as needed.	
Engagement of the local authorities and community allowed to set priorities in training of staff at PHC and community levels, adaptation of existing diabetes management protocols, provision of diagnostic equipment including point of care devices, development of patient files, recall system and context specific health promotion activities in the community and bridging the gap of therapeutic patient education. A review of the programme in collaboration with a local university will allow to plan a moving on strategy.	

In each humanitarian emergency, selection of the NCDs to be addressed should also be based on local disease burden (see example Pakistan, Table 1), needs of the affected population, factors related to the context, and local and agency capacity in providing services. Chronic conditions such as epilepsy, pre-existing severe mental disorders or diseases that are highly prevalent in certain regions of the world (such as sickle cell anaemia) can be added when appropriate. Guidance on mental health exists with the ICRC’s basic psychiatric care package to facilitate provision of care for mental health disorders along with other NCD care. For acute mental illness resulting from the humanitarian crisis, the WHO has developed the mhGAP for humanitarian interventions [21]. In a number of MSF projects, routine screening for depression is included in the care provided to NCD patients, and patients with severe mental illness are managed within the NCD cohort with clinicians trained on mhGAP “author’s personal knowledge”.

**Fig. 1:**
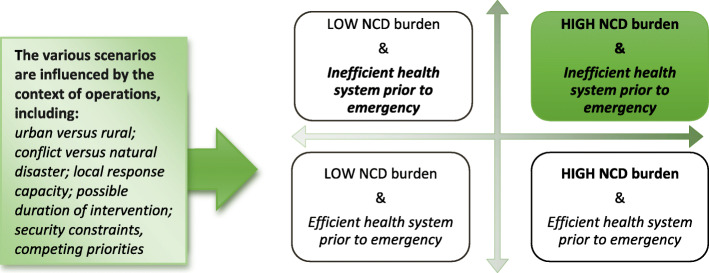
Humanitarian contexts and NCD response planning

The capacity of the health system to provide care to its population prior to the emergency significantly influences the health and recovery of the population during and following an emergency [15]. In preparing for a response, humanitarian actors need to think about the various scenarios influenced by the context (urban versus rural; conflict versus natural disaster versus public health emergency; possible duration of interventions; security constraints; competing priorities; crisis level; NCD burden and local response capacity prior to the crisis (Fig. 1) and how this will impact prioritization.

Some individuals with NCDs are more prone to critical acute exacerbations or to severe consequences with an interruption in care and need to be prioritized to reduce morbidity and mortality (Table 2).

**Table 2:**
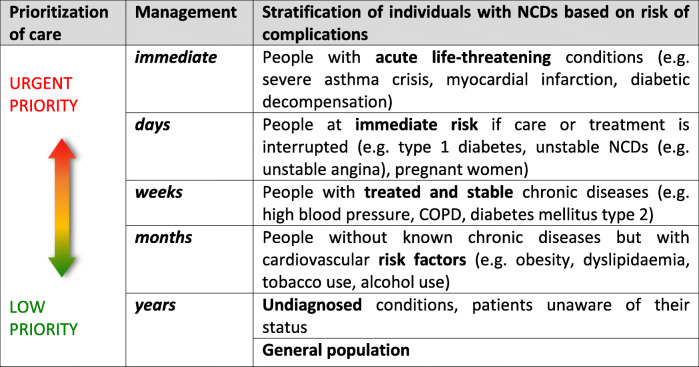
Prioritization of care

For example, an individual with type 1 diabetes is at great risk of serious complications and even death if unable to access insulin for just a few days [22]. Conversely, a person with type 2 diabetes who has been well-controlled on oral hypoglycemic agents is unlikely to suffer any immediate consequences if unable to obtain their oral hypoglycemic for a few weeks, even months. Therefore, in the acute phase of an emergency, a system for triage and prioritization of individuals based on the risk of complications and severity of their chronic disease should be implemented. This approach is particularly important in settings where resources are limited. Other “risk or vulnerability factors”, such as stress, loss of family, loss of livelihood and home, being disabled, extremes of age, etc. could also be considered.

The proposed considerations for the management of NCDs in humanitarian settings are centered on key components, embedded within the overarching principles of Universal Health Coverage (UHC) (i.e. people-centered, accessible, equitable, comprehensive, integrated, accountable and efficient care) [23]. These elements are built on strong ethical principles. The overall humanitarian response to NCDs requires advocacy and research to further strengthen implementation.

### Key components of NCD care delivery

NCD management should ensure continuity of care through an integrated approach at each level of health care focusing on primary care with referral pathways to prevent excess mortality and morbidity. An integrated PHC approach will enable a more sustainable and comprehensive intervention, especially if implemented through local facilities. In absence of a functional local health structure, a reliable health partner should be identified, supported and empowered, where possible avoiding vertical programming [16, 24–26]. However, in alignment with the existing system, NCD management should be supported where it exists, as for example in Libya, where the ICRC is supporting specialised diabetes clinics. In addition, NCD care should be integrated into existing HIV or TB programmes, as these are also addressing chronic conditions. Lessons learnt from these programmes should be used for NCD care including a differentiated service delivery approach. In addition, community engagement can help to empower patients in self-management of their condition and facilitate promotion of healthy lifestyles.

**Fig. 2:**
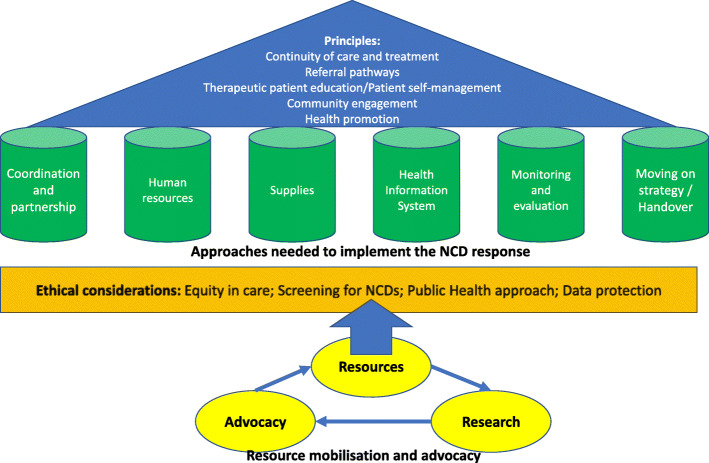
Framework for responders to NCDs in humanitarian settings

The following elements are pillars of the response: coordination and partnership; human resources; supplies; health information system; monitoring and evaluation; and exit strategy/handover. They are equally important and mutually supporting, but need to be adapted during complex emergencies (emergencies where multiple concurrent factors lead to disruption of services [27]), given the context-specific constraints, management challenges, and, at times, the high mobility of affected populations. Ethical considerations such as equity in care, screening or not for NCDs, public health versus individual health approach and data protection need to be addressed from the onset of an NCD programme in addition to advocacy and resource mobilization (Fig. 2).

#### Coordination and partnership

The response to NCDs includes identification of the intervention gaps in NCD management, the most urgent needs and the available and functioning resources (human, financial, infrastructure, etc.) in the given context. It should be in congruence with the national health system and integrated into existing services when possible. This approach will encourage ownership and facilitate the future handover of activities to local health services. Similarly, partnership with other international actors and with local non-governmental organizations involved in NCD care should be ensured. During the needs assessment, defined as the systematic evaluation of needs of a population and responses required [28], it will be important to assess the presence of these local actors and resources that remain functional following the crisis. Not all stakeholders will provide the whole range of interventions and health services. The efficient use of available resources and expertise to equitably address the needs and gaps in the NCD response should be discussed.

In addition, other local resources, such as HIV and TB programs, community groups, Women’s groups, Elders and Diabetes associations, National Red Cross and Crescent Societies working in and for communities through a support network are key partners with whom to define priorities, to assist with the management of NCDs during the crisis and to provide continuity of support post-crisis [29–31]. These organizations might have existing resources such as patient education programs or support networks.

#### Human resources

Staff required for the management of NCDs include nurses, general medical practitioners, health promotion staff, and community health workers. The complex nature of many NCDs can lead to longer consultation time requirements than those for acute illnesses, which should be factored in when determining the number of staff required. If local human resources lack experience or expertise in NCD management, they may need to be supported by externally-sourced expert staff, and the treating medical team will need access to specialist support for challenging cases, either through patient referral or remote support to the clinicians. Standardised training material and context-appropriate guidelines should be made available to enhance staff knowledge and skills for NCD management. Regular supportive supervision and evaluation of staff performance should be set up.

In HIV programs, resource allocation has been optimized through task-shifting and sharing, such as using nurses to follow-up stable patients [29]. This approach is increasingly being used in NCD care and should be routinely considered in humanitarian contexts [32]. For example, in many MSF programs doctors manage treatment initiation, and nurses provide routine treatment continuation for stable patients [33–35] . Patient education can also be shared among different team members, and health promotion staff (e.g. community health workers/volunteers/peers) can be used for group education.

#### Supplies

Care for people with NCDs requires reliable, affordable and regular access to medical supplies (i.e. essential medicines, laboratory tests, and medical devices such as glucose meters) to avoid treatment interruption or inappropriate management. A strategic plan for provision of supplies should be established in the initial step of the response and should include the following elements:

- A list of medical supplies based on the agency-prioritized interventions, the WHO model list of medicines, the Package of Essential Noncommunicable Diseases (PEN) list for devices and the national essential drug list.
- A system for procurement and storage of supplies.
- Cold chain capacity. This aspect can be coordinated with other agencies providing services requiring cold chain such as immunization.
- A buffer stock in the event of an interruption in the supply chain, a rapid displacement of the services for security reason, or a massive increase in the needs for NCD care.

The plan should be regularly monitored and evaluated, and be adjusted to evolving needs.

The selection of medication lists should take into account local medication availability to facilitate handover of patients to local authorities and/or the WHO model essential medicines list [36]. Local Market Assessments (LMAs) and/or Model Quality Assurance System assessments (MQAS) may need to be conducted/commissioned to validate quality sourcing, storing and distribution practices of quality assured medicines. Maintenance during an acute emergency of expensive complex medical regimens might not be possible. The use of quality-assured generic medicines is recommended for safety and cost-effectiveness, but attention should be paid to minimizing medication changes that may induce patient confusion or non-adherence, particularly in short-term response. In insecure settings, stockpiling of medicines at health facility level and provision of “run-away” kits to patients avoids treatment interruption and consequent complications. The 2017 version of the WHO Interagency Emergency Health Kit (IEHK) includes some medicines for patients presenting with acute exacerbations of NCDs, and a new NCD kit for the management of acute and chronic NCDs is currently being piloted [37].

In addition to medicines, diagnostic tools are needed. The choice of required equipment will be based on the context and needs assessment as for medicines (including capacity of staff to interpret results). It may be possible to use existing laboratories, if they have reliable supply and quality control mechanisms, as is done in an ICRC-supported project in Iran where patients are provided with vouchers. A number of relevant point of care tests exist and should be considered. For example, the provision of HbA1c point of care devices to some MSF PHC clinics has allowed for more timely and reliable adjustment of treatment for diabetes [37]. However, there is a lack of easy, inexpensive laboratory devices at PHC level that include all key cardiometabolic markers, e.g. glucose, HbA1c, lipids, creatinine, liver enzymes.

For new laboratory equipment, the following points should be considered:

- Local vs international purchase, particularly regarding maintenance and renewables/spare parts supply and preferences of the national system (for sustainability reasons)
- Importation and licensing
- Quality assurance
- Options for maintenance
- Supply of renewables and spare parts
- Ability to hand over

#### Health information system

Where possible data collection for chronic diseases should include more parameters than the aggregate consultation numbers and diagnoses that are typically collected in humanitarian settings. Information on cohort size, patient outcomes, and co-morbidities enables better monitoring of the intervention. Individualized data collection is also beneficial for patient management. As an example, United Nations Relief and Works Agency for Palestine Refugees (UNRWA) has implemented an electronic real-time patient record allowing for patient and cohort monitoring [38].

Furthermore, a strategy allowing the patient to have access to their key medical information independently of their mobility should be developed as described above. Patient-held clinical records or a patient passport should be used to promote clinical continuity and systematic care in highly mobile populations and those in insecure settings. As a simple solution, in some settings where patients are expected to move, MSF provided patients with a patient passport or a digital photograph of their medical file, enabling them to store their key medical information with them [39]. Patient-held records should include context-adapted comprehensive patient education messages. As discussed below, data protection systems should be in place for any data tool that is used.

Information needed and the systems for collecting it are presented in Table 3. The systems should be well interlinked to avoid duplication of data collection where possible. The patient data should feed into the facility data, which in turn should feed into the programmatic and operational data and overall monitoring and evaluation.

**Table 3:** Data collection systems

Information level	Type of system	Indicators
Patient	A patient file A patient held record	Data to inform clinical care, follow up, and individualized data points. Specific clinical information to help patient care management when provided in different settings.
Clinic	Register held at the facility or electronic database	Data to inform the management of the day-to-day clinic activities, call and recall system, and to improve services quality.
Program (HIS)	An electronic database for operational use	Data to inform with overall programmatic planning, management of resources (medicines/consumables, human resources, etc.) as well as reporting surveillance and monitoring and advocacy.

#### Monitoring and evaluation

The data collected should be used for monitoring and evaluation purposes, as is done in the cohort monitoring of refugees with diabetes in Jordan [40]. Monitoring is a continuous process that allows the identification of shortcomings and gaps in the delivery of the interventions in order to adjust the activities accordingly, by assessing the inputs, the activities and the outputs of the interventions. It also serves to improve accountability to the beneficiaries and other stakeholders by objectively showing the used and available resources, the potential delays, and the achieved results. In the field of NCD interventions, this implies monitoring patient outcomes, comorbidities, prevalence of NCD risk factors, proportion of patients receiving treatment and the overall response to NCDs by all stakeholders among other indicators to gather. Evaluation should be performed periodically (every 3–6 months) to assess the quality, the outcomes and the impact of the response delivered, and can include a clinical audit to assess quality of care provided [41]. A list of NCD indicators for both monitoring and evaluation purposes is currently under development by humanitarian organizations.

#### Moving on strategy/handover

As continuity of care is critical for adequate NCD care, a handover strategy should be considered from the start of the operation. Integration of the response into primary health care delivery will help in this regard, above all if implemented through existing local facilities when functional. In the absence of existing local capacity, identifying and supporting a local or international healthcare provider to take over NCD care after withdrawal is important. Vertical programming should be avoided. If the intervention was set up rapidly without clear objectives or handover strategy, a review process within the first year should be carried out. Depending on the findings, the project should be adapted to reflect any revised objectives and/or long-term plans, including integrating a moving on/handover strategy if the plan is to pull out after the crisis.

Given the fact that NCD management in emergencies is not yet a routine part of humanitarian response, it is recommended to implement the suggestion of Slama et al. [4] for a comprehensive debriefing of all stakeholders, aiming to share lessons learned and increase preparedness for future emergencies. Accountability to beneficiaries needing lifelong care requires planning withdrawal from support of international organizations with adequate handover to local authorities and / or relevant health actors. In case the authorities fail to resume their responsibilities fully, potential interested organizations are approached to take over fully or partially the support of the provided services.

### Continuity of care and treatment

As with episodic illnesses, NCDs may require the management of acute, life-threatening or uncontrolled conditions. However, a distinctive feature of these varied diseases is their need for continuity of care, including the detection and monitoring of disease progression (follow up care) and the management of long-term complications. This implies regular access to care, medicines, medical devices, and laboratory facilities for diagnosis and monitoring, and referral when necessary to specialized services. Patients should also be guided, supported and monitored for treatment adherence and side effects. Important secondary prevention activities (such as regular foot care in diabetes patients and aspirin after myocardial infarction) should be implemented as part of routine care, together with therapeutic education.

The challenge in humanitarian contexts is the uncertainty of following up individuals for care and monitoring. This aspect should be regularly evaluated during the response to ensure the development and implementation of an adaptive follow-up system. This will ensure that patients are continuing to receive care independently of their mobility. Patients in transition, particularly those in migration, should have personalized contingency plans for their NCD management and patient-held records (see above 3.4 health information system). This can include provision of information about possible access points where they could consult along the way and/or provision of larger quantities of medication to cover the periods during which patients do not have access to a health facility or NCD medications.

Follow-up care should be provided to all patients. However, as noted above, different risk profiles exist among patients with NCDs. Based on disease stability, complexity and risk of complications, the interval of follow up will change. Patients with uncontrolled NCDs or recovering from an acute condition may require visits monthly or more, whereas those with stable and controlled diseases may only require trimestral or bi-annual clinical review visits.

#### Referral pathways

NCD care and patient management strategies should be centered at the PHC level using the WHO PEN model [42], integrated with other health care services. The management of acute conditions beyond initial stabilization (e.g. dialysis, myocardial infarcts, or stroke) requires referral pathways to secondary and tertiary level care centers. These should be rapidly put in place, based on local availability and context. The initial needs assessment should provide the necessary information on which health services and transport systems are available locally. Supporting patients with out-of-pocket costs - including transport - should be considered. Further guidance on referrals can be found in the UNHCR “Principles and Guidance for Referral Health Care for Refugees and Other Persons of Concern” [43].

#### Therapeutic patient education/patient self-management

Besides medical treatment, people with NCDs require information, education and tools to assist in the management of their NCDs. Therapeutic patient education promotes patient self-management and empowerment, both of which are key components of the management of any chronic conditions [44].

Topics such as health literacy, treatment adherence, medication side effects, and healthy lifestyles should be discussed, adapted to the context and patient. Patient education should be provided at each visit during the response. When resources are limited and in the early stages of the humanitarian intervention, health care providers can give standardized messages covering basic disease literacy and adherence advice. Later in the response, education can be expanded according to the patient’s specific needs, potentially supported by other actors. For instance, community activities or NCD support groups are good avenues to deliver educative messages and can be done in collaboration with the community. Patient education is particularly important in the case of a mobile population where patients frequently change health services and providers. In addition, small booklets with pictograms and simplified explanations on treatment and recommendation for the next appointment could be used. Along with patient education, devices such as glucose meters are crucial for home and self-care management and adherence.

#### Community engagement

Community engagement is key to NCD management, as it allows the identification of community needs, priorities and local capacity, understanding of the context, and proximity to the population. Involving persons of the affected community in decisions puts them in the center of an intervention answering their needs (see Table 4). Regarding care for NCDs it also enhances continuity of care, prevents overburdening of health structures, and promotes affected people’s autonomy and support. Community health outreach can be provided by community health workers or NCD community leaders to promote behaviour change (healthy lifestyle), give information on available services and links to ongoing clinical care, and provide follow up care (e.g. supervision of therapy and patient education) [45].

**Table 4:** **Iran Mashhad** : to illustrate tailored health promotion, beneficiary engagement, accountability to affected populations

In Mashhad, Iran, the PHC services collaborate closely with representatives of the community to understand the constraints, so as to adapt the intervention to people’s needs and capacities. Health promotion and nutrition advice are tailored to persons with low income who can hardly afford to buy healthy food. Regular visits by the nutritionist and social worker to the market and discussions with shopkeepers and beneficiaries allow to identify appropriate affordable and acceptable season food items.	

#### Health promotion and healthy lifestyle support

Population-based lifestyle prevention is not a priority in emergency settings, but there may be opportunities to leverage patient-targeted messages to have a broader impact, such as giving context-adapted lifestyle messages in waiting rooms and communities so that patients’ families and communities can also benefit. This can be facilitated by linking with any existing community programs and be integrated into outreach activities. In some settings patients will have limited control over lifestyle issues, and targeted advocacy for a facilitating environment may be important, such as banning indoor smoking. Healthy nutrition is very important for cardiovascular disease and diabetes management and nutrition advice can be integrated in the package of provided services (see example Iran, Table 4). However, populations in humanitarian settings may be dependent on food aid. In this case, links with organizations providing food supplies are needed to promote the selection of balanced nutrients in food aid, or to propose cash and vouchers to allow the purchase of specific food groups. Ensuring that the supplies do not contain excessive sugar, oil and salt can provide a population health benefit as well as addressing the needs of specific patient groups. Community gardening, for example, where people grow their own fruits and vegetables would provide them with a physical activity, improve their nutrition and empower them personally, potentially benefiting their mental health [16].

Specific NCD patients, such as pregnant women, children or those with type 1 diabetes should be targeted for support of appropriate food rations from the initial phase of an intervention.

New technologies including digital health promotion have shown some promise in humanitarian settings. For example, mHealth was introduced in selected PHCs in Lebanon to facilitate counselling about lifestyle behaviours by health care providers [46]. However, these solutions need to be adapted to the local context and the use of digital tools by the population, as well as include sufficient data protection measures (see section on data protection).

### Ethical considerations

Beyond the practical aspects of implementing a response to NCDs in humanitarian emergencies, there is also the need to consider a variety of ethical issues. Access to health for persons affected with an NCD is part of human rights, which are universal. A humanitarian emergency should not be an excuse to neglect the provision of NCD care.

#### Equity in care

When providing access to health services for Internally Displaced Persons (IDP) and refugees, it is essential to ensure similar services are available to the resident or host population [47], which may also endure a disruption of routine health care activities [48, 49] and overburdened health services [50]. For example, this was seen in Mali when different populations with diabetes (IDPs, refugees, and the host population) all faced challenges requiring distinct approaches [50]. Strengthening existing health services will benefit host community, as well as refugees and IDPs inside areas of functioning health systems.

#### Screening for NCDs

As in non-humanitarian settings, any screening program should have an evidence base which supports its impact and cost benefit, and should consider the ability for individuals to access treatment. Consideration should also be given to not overburdening already stretched health services, and to implementing mechanisms for addressing false positive results. Screening at the population level has health system, economic and ethical implications and is not usually part of a humanitarian response. Targeted screening for risk factors or comorbid NCDs in patients with known chronic conditions (including HIV and TB) is clinically important and should be done when possible, taking into consideration individual, health system and cost-effectiveness implications and availability of treatment [51].

#### Public health approach

A public health approach is frequently required during a humanitarian emergency. This concerns allocation of resources to most efficient interventions benefiting the largest number of patients. As previously discussed, an example may be the use of generic instead of expensive regimens. Unless it is the focus of the intervening agency, management of more complex cases (e.g. cancer, renal failure) is usually not feasible, cost-effective compared to other interventions, or sustainable. Alternatively, referral pathways to care (for example to areas in the country not affected by the emergency or abroad) or supportive palliative care should be proposed.

#### Data protection

Populations affected by humanitarian crises are particularly vulnerable. Their protection, including data confidentiality, is a high priority. Humanitarian actors, in order to be effective, collect often highly sensitive, identifying information including full names and medical data. Medical data is sensitive and must be protected against unauthorized disclosure or use. This requires safe storage and transmission, and other measures in line with data protection principles [52]. Data protection needs to be guaranteed at each step of its collection, from data protection by design, to legitimacy for data processing, to data minimization, data retention, data flow between different entities, data transfer (same organization, different organizations, between organizations and third parties) and data security (ensure that any party having access to data is bound by contractual clauses to data protection).

Standards for data protection, such as the European General Data Protection Regulation (GDPR), and the EU Data Protection Directive (1995/46/EC), the Draft Recommendation on the Protection of health-related data of the Council of Europe consultative committee T-PD (2016) should be considered as benchmarks for data confidentiality. Organizations remain subject to the legislation of countries where they operate. This may pose challenges if health records or medical data are under the authorities of the country of operation. It is essential to ensure that persons keep control over their own data and are informed about their rights [53].

### Resources mobilization, advocacy and research

Funding for NCD care is often still deficient for humanitarian responses, and advocacy in this regard is challenged by the lack of supportive evidence on the burden of disease and impactful response [13, 54].

#### Resource mobilization

Data on costing of humanitarian NCD responses remains limited to date [55]. Costing studies are required in order to identify the most efficient approach to cover the needs of the population as well as mobilize the appropriate resources. Funding for NCD care in emergency settings should be included in the budget of general health assistance programs, reflective of the needs regarding human resources, continuous medication supply, and specific equipment. The short-term nature of humanitarian program funding can prove challenging to the management of chronic diseases, and funders need to consider this in their support. Regular evaluation of the needs can help with supplementary fund raising and budget adjustment. Coordination with other actors on the ground is essential to optimize resource sharing.

As discussed, prioritization of NCD service components is helpful for the efficient use of available funds. When resources are limited, they should also target the patients that are the most likely to benefit due to high risk of complication if care is interrupted. A lesson learnt from the Haiti earthquake is to base interventions not only on needs, but also on capacity and context [56]. Donors should be encouraged to give cash rather than assistance in kind, which may be inappropriate, and not sustainable.

#### Advocacy and research

It is acknowledged that a comprehensive response to management of NCDs is often neglected in humanitarian responses [4]. These operational considerations represent a constructive step towards progress in this regard. However, more efforts are required to improve the capacity to address the needs of increasing numbers of people worldwide affected by both NCDs and emergencies. As the global prevalence of NCDs increases, further advocacy is needed to increase financial resources available to ensure that NCD care can be routinely integrated in essential primary care in emergency contexts. This will require both immediate and longer-term commitment from donors.

To foster increased funding, more evidence on cost-effective interventions and best practices is necessary. This implies a need for more operational research as well interagency sharing of experience on NCD management in emergencies and creative solutions for service delivery in unstable contexts. Most interventions are based on evidence from LMICs and have not been tested in emergencies. Nevertheless, research on NCDs in emergencies implies multiple challenges that need to be addressed, including security constraints, challenges in follow-up, data protection, other urgent needs and capacity of field teams to conduct research.

## Discussion

**Fig. 3:**
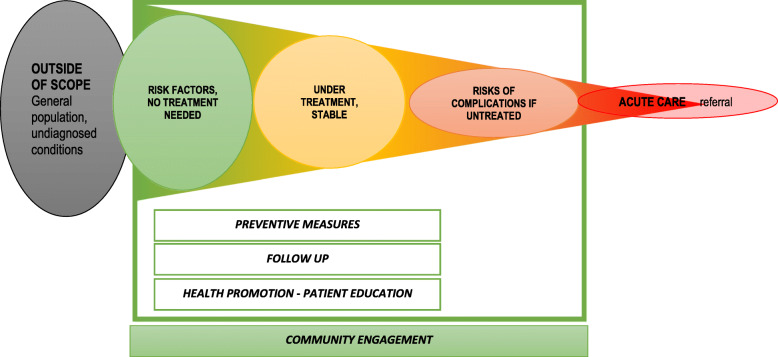
Scope of interventions Adapted from Managing projects adderssing non-communicable diseases: Operational guidelines for field staff. ICRC, Dec 2019 [24]

NCD-related needs of affected populations and the types of resources required vary depending on the context. Any humanitarian emergency will cause disruption to the provision of health services for those with NCDs. With the large number of individuals facing humanitarian emergencies in parallel to the COVID-19 pandemic, NCDs require specific attention by humanitarian actors as well as other global actors as the global NCD response has failed to effectively respond to the needs of these vulnerable populations [23]. We identified the following challenges in developing operational considerations: a lack of evidence and global guidance for humanitarian settings and NCD management; weak existing responses to the NCD burden even in non-humanitarian settings; each humanitarian context having its specificities, with the need to take into account the population, existing services, and priorities of different stakeholders; organizational and operational considerations of different humanitarian actors; and overall finding a consensus on an overarching approach that is relevant to the different contexts and actors. The model proposed (Fig. 3) highlights the main components and approaches to be considered.

Key components include access to treatment, continuity of care including referral pathways, therapeutic patient education/patient self-management, community engagement and health promotion. In order to implement these components, a standardized approach will support a consistent response, and should be based on an ethical foundation to ensure that the “do no harm” principle is upheld. Advocacy supported by evidence is important to generate visibility and resource allocation. Although not included in these guidelines, inclusion of NCDs in agency preparedness plans is also an important facilitator for NCD response capacity.

Beyond this, as NCDs encompass a spectrum of diseases and care requirements, some form of prioritization is needed based on available resources and the guiding principle of “do no harm”. Focus on what is feasible also needs to consider the feasibility during different stages and types of humanitarian emergencies. In the acute phase of an emergency, a system for triage and prioritization of individuals based on the risk of complications and severity of their chronic disease should be implemented. To date such considerations have been missing to guide the humanitarian response for NCDs as well as global guidance on NCDs negating the specificities of humanitarian settings [23].

## Conclusion

Although humanitarian actors are increasingly gaining experience in the feasibility of including NCD care in their emergency response, structured guidelines have been missing. The aim of these operational considerations is to provide guidance on the wide range of issues facing humanitarian actors on the ground with management of NCDs and provide a series of elements to guide the required response. The process of elaboration and exchange of experience behind this paper has now served as a basis for the development of operational guidelines for the ICRC [16], IRC and UNHCR [57] which have also been field tested.

## Data Availability

The full operational guidelines are available on request from the authors.

## Abbreviations

COPD: Chronic Obstructive Pulmonary Diseases
GAP: Global Action Plan
GDPR: General Data Protection Regulation
HIV: Human Immunodeficiency virus
ICRC: International Committee of the Red Cross
IFRC: International Federation of the Red Cross
IEHK: Interagency Emergency Health Kit
LMAs: Local Market Assessments
LMIC: Low and middle-income countries
mhGAP: Mental Health Gap Action Programme
MQAS: Model Quality Assurance System
MSF: Médecins Sans Frontières
NCD: Non-communicable diseases
PEN: Package of Essential Noncommunicable Diseases
PHC: Primary Health Care
TB: Tuberculosis
UHC: Universal Health Coverage
UN: United Nations
UNHCR: United Nations High Commissioner for Refugees
